# Association of soybean-based food with the prevalence of anaemia among reproductive-aged men and women in rural Central Java, Indonesia

**DOI:** 10.1017/S1368980021005000

**Published:** 2022-12

**Authors:** Callum Lowe, Haribondhu Sarma, Matthew Kelly, Johanna Kurscheid, Budi Laksono, Salvador Amaral, Donald Stewart, Darren Gray

**Affiliations:** 1 Australian National University, Acton 2601, Australia; 2 Swiss Tropical and Public Health Institute, Basel 4051, Switzerland; 3 Universitas Diponegoro, Semarang 50275, Indonesia; 4 Griffith University, South Brisbane, QLD, Australia

**Keywords:** Anaemia, Soybean, 24-hour food recall, Dietary assessment, Soil-transmitted helminths, Indonesia, Nutrition

## Abstract

**Objective:**

To assess the association between soybean consumption and anaemic status in Central Java, Indonesia.

**Design:**

As part of an overarching sanitation improvement intervention in Central Java, Indonesia, we conducted a cross-sectional study in four rural villages. The study consisted of a 24-h food recall, anthropometric measurements, blood Hb measurement and stool sampling to test for soil-transmitted helminth (STH) infection status. A binary logistic regression model was fitted to test the association between soybean consumption and anaemic status after adjusting for socio-demographic factors, STH infection, dietary diversity and anthropometric status.

**Setting:**

This study took place in four rural villages of Wonosobo regency, Central Java, Indonesia.

**Participants:**

Participants were rural villagers aged between 15 and 49 years.

**Results:**

A total sample size of 763 was attained, of which 231 were anaemic. The prevalence of anaemia was 30·2 % among men and women of reproductive age, and highest among young males. Consumption of soybean was high (79·8 %). After adjusting for covariates, the protective association between soybean consumption and anaemia was statistically significant (AOR = 0·53, 95 % CI = 0·30, 0·95, *P* < 0·05). There was a positive association with anaemia among underweight (AOR = 2·75, 95 % CI = 1·13, 6·69, *P* < 0·05) and those with high diet diversity (AOR = 1·40, 95 % CI = 1·00, 1·97, *P* < 0·05).

**Conclusions:**

Our results were consistent with studies from other countries finding a protective association between soybean consumption and anaemia. This association appeared stronger for tofu than for tempeh. The prevalence of anaemia in rural Central Java is relatively consistent with nation-wide statistics indicating that interventions targeting anaemia are still largely required.

Anaemia is a condition where Hb concentrations are below the normal level to meet a person’s physiological needs, and affects one-third of the world’s population^(1)^. Anaemia can cause compromised immune system function, weakness and cognitive dysfunction^(2)^. Whilst the causes are multi-factorial, there is increasing awareness of the contribution of helminthiases^(3)^. Fe-deficiency anaemia (IDA) caused by nutritional Fe deficiency is the most common cause of anaemia^(2)^ and a national problem in Indonesia^(4)^. Over the last decade, the prevalence of anaemia in Indonesia has steadily increased^(5)^ following a downward trend observed between 1997 and 2008^(6)^. Limited literature exists on the causes of this increase, although it is possible that the rising rates of overweight/obesity are responsible as these conditions are also associated with anaemia^(7)^. In 2018, the prevalence of anaemia among pregnant women nationwide was estimated at 48·9 %^(6,8)^. No population-wide estimate of anaemia prevalence across all demographics in Indonesia has been reported in over a decade, but more recent studies still report high prevalence, with one 2016 study finding 40·7 % of pregnant Indonesian women in North Sumatra anaemic^(9)^.

Pregnant women represent one of the most at-risk groups for anaemia because of the increased requirements of Fe during pregnancy^(10)^. One of the major interventions that is part of national policy in Indonesia towards IDA is Fe supplementation for pregnant women and wheat flour fortification with Fe^(11)^. The effectiveness of Fe supplementation however has been compromised. Two major obstacles have been faced – not meeting supplementation frequency and amount requirements among young women^(12)^ and discontinuation of supplement use among Indonesian women during pregnancy^(11,13)^. Among adolescent girls, anaemia is of greater risk due to the onset of menstruation and increase in muscle mass and blood volume^(14–16)^. This is of great concern in Indonesia whereby misinformation surrounding the risks and prevention of anaemia has been reported by adolescent girls and their parents^(17)^. Often forgotten however is the danger of anaemia among men, where similar symptoms that appear in women such as lethargy and fatigue are common, but are also thought to contribute to decreased economic productivity^(18)^. Fe supplementation in Indonesia is only recommended for pregnant women, and so other demographics such as men are not subject to these direct interventions.

Factors associated with anaemia commonly observed include low socio-economic status^(19)^, low education^(19–22)^, discontinued use of Fe supplements^(21,22)^, low dietary diversity^(20,23)^ and helminth infections^(24)^. Hookworm infection contributes to anaemia as it feeds off its host’s blood causing blood loss^(25)^. In Indonesia, the association between hookworm infection and anaemia has been observed (OR = 2·6, 95 % CI = 1·2, 5·8, *P* < 0·05)^(24)^. Low dietary diversity can mean low consumption of bioavailable Fe as well as deficiency of minerals and vitamins that increase the bioavailability of Fe. This is supported by a study among adolescent girls in West Java, Indonesia, that found lower consumption of meat, chicken and fish as well as lower diet quality was associated with anaemia^(26)^. While animal foods such as those mentioned provide a rich source of bioavailable Fe^(27)^, meat consumption in Indonesia is the 16th lowest in the world and lower than other south-east Asia countries^(28)^.

Soybean is a high Fe containing legume^(29)^ and is a staple in the Indonesian diet^(30)^. Due to its elevated Fe content, the association between soybean and soybean-based food consumption with anaemia has been explored in much literature^(29,31–34)^. Pregnant women consuming an optimised diet with fermented soybean and vitamin C-rich fruit (vitamin C increases absorption of Fe^(29)^) in Indonesia have been demonstrated to experience a smaller decrease in serum Hb concentration during pregnancy^(29)^. Among Chinese adults, the consumption of tofu, a soybean-based product, is associated with reduced odds of anaemia (AOR = 0·30, 95 % CI = 0·20, 0·47)^(32)^. Little research however has been conducted to evaluate this association in Indonesia, despite wide prevalence of anaemia and soybean consumption. The two major soybean products consumed in Indonesia – tofu and tempeh which make up 70 % of soybean consumption^(30)^ – have not yet been studied together in relation to anaemia, and the existing consumption of them needs to be quantified to assess their potential role as part of an anaemia intervention.

In the Indonesian context where the prevalence of anaemia is rising, other public health interventions need to be introduced alongside Fe supplementation to curb this rise. The identification of simple, culturally acceptable foods may aid existing interventions. Given this situation and the aforementioned gaps in the literature, our study aimed to assess the association between consumption of soybean-based food, the prevalence of anaemia and interactions with STH infection status among rural villagers in Central Java, Indonesia.

## Methods

### Study design and sample

This nutrition study is part of an overarching sanitation improvement intervention in rural Central Java, Indonesia. A cross-sectional study design was employed where a 24-h food recall was conducted and anthropometric and blood–Hb measurements were taken. Participants in the study were from four rural villages in Central Java, Indonesia. These villages are located on the outskirts of the district town Wonosobo and on the foothills of neighbouring mountains Mt. Bismo and Mt. Sindoro. This overarching study was a community-based cluster-randomised intervention trial where eight villages were selected on the basis of low levels of household latrine ownership, and stratified by topography (four mountain Dusun and four hillside Dusun). The villages were further stratified into control and intervention groups. The four villages selected for this study comprised the control villages for the intervention. Data collection was carried out in November–December 2018. From each village, 110 households were randomly selected and all members of those households aged 3 years and over were invited to join the study. After the 1-year follow-up of the study, participants in control villages were approached again and invited to take part in the nutrition survey. At baseline, a total of 1876 participants took part in the sanitation improvement intervention. At follow-up, a total of 1521 participants from the four control villages took part in the nutrition survey.

### Data collection

Dietary intake data was collected from a 24-h food recall. For this study, only participants aged between 15 and 49 years old with complete data were used in analysis because of the heightened risk of anaemia among adolescents and women of reproductive age^(10,14–16)^. This resulted in a final sample size of 763 participants. The 24-h food recall was a semi-structured questionnaire administered by trained data collectors. Data collectors were local health staff with knowledge of locally relevant food types. Participants were asked to provide a recount of the foods and beverages they consumed in the past 24 h including the amounts (number of cups, pieces or glasses where relevant), side-dishes, method of preparation (e.g. purchased or home cooked, fried, steamed) and the ingredients used.

Anthropometric indicators comprising height (cm) and weight (kg) were also measured by trained project staff. Height was measured using a portable stadiometer with a measuring range of 150–2, 100 mm and a graduation of 1 mm. Participants were asked to take off their shoes and stand with their back straight against the stadiometer. Weight was measured with an Omron digital weight scale with a 0·1 kg increment. Participants were asked to remove shoes and jackets for an accurate weight measurement.

A finger-prick test for blood Hb concentration was performed using *Hemocue 201 (Angelholm, Sweden)* instruments by trained data collectors. Using a pre-prepared lancet, participants’ fingers were pricked, and the first two drops of blood were wiped away. Following this, light pressure to the finger was reapplied to draw blood into a microcuvette. The microcuvette was placed in the Hemocue analyzer and the Hb measurement was recorded.

### Assessment of anaemic status

Anaemic status was determined using WHO cut-off points based on blood Hb concentrations^(35)^. Blood Hb concentrations were converted to g/l and placed into categories (Non-Anaemia, Mild, Moderate, Severe) for each participant based on the cut-off values from the appropriate age/sex stratum. Adjustment for elevation was not required and smoking status was not measured. Mild, moderate and severe anaemia were grouped as anaemic to create a binary outcome variable. As a result, anaemic status is considered as Hb concentration in g/l below 120 for non-pregnant women (aged 15+), below 110 for pregnant women and below 130 for men (aged 15+)^(35)^.

### Measurement of dietary variables

Soybean was in all but two instances consumed in the form of Tofu or Tempeh. As such, soybean consumption was categorised as ‘Neither’, ‘Tempeh only’, ‘Tofu only’ or ‘Tempeh and Tofu’. Tofu and Tempeh had to be a main component of dishes/snacks to be positively classified. Common examples included fried/steamed tofu or tempeh and vegetable dishes made with tempeh and tofu. Diet diversity was measured using the FAO minimum dietary diversity for women guidelines^(36)^. This involves categorising food into ten distinct categories (Grains/white roots and tubers/plantains, Pulses, Nuts and seeds, Milk and milk products, Meat/Poultry/Fish, Eggs, Dark green leafy vegetables, other vitamin A-rich fruits and vegetables, other vegetables and other fruits). High diet diversity was defined as having consumed foods from at least five of these categories, otherwise classified as low diet diversity.

### Measurement of BMI

BMI was calculated by dividing weight in kilograms by height in metres squared. For adults aged 20+ years old, BMI cut-off for Asian populations was employed to categorise individuals as normal (18·5–23), underweight (< 18·5) and overweight or obese (> 23)^(37)^. For adolescents aged between 15 and 19 years old, BMI for age z-scores was calculated using WHO AnthroPlus software^(38)^. Overweight or obese was defined as Z > 1 sd and underweight as Z < –2 sd ^(39)^. Outliers for BMI were considered where BMI for age z-scores were < –5 or > 5 for children, and BMI < 14 for adults.

### Soil-transmitted helminth infection status

Infection with soil-transmitted helminths was determined by analysis of stool samples as part of the overarching sanitation improvement intervention. Participants were approached and asked to provide stool samples that would be collected the next morning. Participants were provided with a sealable plastic container. Parents were instructed on how to obtain stool samples from young children. At the time of collection, stool samples were placed into two centrifuge tubes – a 2 g sample of faeces was shaken vigorously with 8 ml of 10 % formalin solution, and a 3 g portion was shaken vigorously with 3 ml 5 % potassium dichromate. Waste was collected in a clinical waste bag and disposed of appropriately. Centrifuge tubes with stool samples were stored at room temperature away from direct sunlight during storage and transportation to Poltekkes Kemenkes Semarang (Semarang Ministry of Health Polytechnic) for analysis. The identification of STH eggs was performed using the faecal flotation method^(40)^. Species identified were Hookworm, *Trichuris trichiura* and *Ascaris lumbricoides*.

### Statistical analysis

Statistical analysis was performed in IBM SPSS version 26. The socio-demographics of the study population was tabulated and the percentage of participants within each socio-demographic category consuming soybean was calculated. Following this, the prevalence of anaemia by socio-demographic and nutritional-related variables was calculated, and crude odds ratios were calculated for anaemic status. Mean Hb values by age/sex and anaemic status were tabulated. A binary logistic regression was fitted to model the association between soybean consumption and anaemia. Associations between soybean consumption, BMI and diet diversity with anaemia were measured after adjusting for age, sex, education and village in the first model, and then all covariates were mutually adjusted in the second model.

## Results

The demographics of the study population and associations between socio-demographic and nutrition factors with soybean food consumption are presented in Table 1. The study population was roughly evenly split between males and females as well as across all four villages. Few participants were underweight (4·6 %) and almost a quarter (23·8 %) were overweight or obese combined. Approximately 80 % of the study population consumed soybean products. Little variation in consumption was observed between socio-demographic factors, although there was some indication that tempeh consumption increases with age. Tofu consumption was less common (39·5 %, sum of Tofu only and Tofu and Tempeh) than tempeh consumption (67·1 %, sum of Tempeh only and Tofu and Tempeh).

**Table 1:** Associations between socio-demographic and nutritional factors with soybean consumption

				Soybean consumption							
						Yes					
						Tofu only		Tempeh only		Tofu and Tempeh	
Study sample		*n*	%	No *n*	%*	*n*	%	*n*	%	*n*	%
All		763	100	152	19·9	100	13·1	311	40·7	202	26·4
Sex	Male	378	49·5	74	19·6	45	11·7	153	39·7	113	29·4
	Female	385	50·5	80	20·8	55	14·5	158	41·6	89	23·4
Age (years)	15–19	121	15·8	27	22·3	18	14·9	45	37·2	31	25·6
	20–29	199	26·0	48	24·1	25	12·6	77	38·7	49	24·6
	30–39	215	28·1	34	15·8	32	14·9	89	41·4	60	27·9
	40–49	230	30·1	43	18·7	25	10·9	100	43·5	62	27
Village	Topengan	182	23·9	36	20·1	30	16·8	67	37·4	46	25·7
	Moyosari	216	28·3	45	20·7	27	12·4	97	44·7	48	22·1
	Rajon	161	21·1	37	22·7	14	8·6	66	40·5	46	28·2
	Losari	204	26·7	34	16·5	29	14·1	81	39·3	62	30·1
Education	No school	57	7·5	8	14·0	9	15·3	23	39	20	33·9
	Elementary	327	42·9	63	19·3	41	12·6	141	43·4	82	25·2
	Junior Secondary	232	30·4	49	21·1	34	14·8	86	37·6	60	26·2
	Senior Secondary	112	14·7	26	23·2	14	12·1	42	36·2	33	28·4
	College, or higher	35	4·6	8	22·9	2	5·6	19	52·8	7	19·4
Anthropometry	Underweight	35	4·6	9	25·7	2	8·7	10	43·5	4	17·4
	Normal	544	71·6	109	20	56	13	178	41·2	117	27·1
	Overweight/Obese	181	23·8	35	19·3	42	13·5	123	39·7	81	26·1
Soil-transmitted helminth infection	Yes	75	14·8	16	21·3	11	14·7	28	37·3	20	26·7
	No	431	85·2	82	19·0	54	12·6	191	44·4	103	24

* Percentages expressed as proportion of participants in soybean consumption categories.

The distribution of mean Hb measurements by age/sex group is presented in Table 2. Mean Hb concentration was lower among women of all age groups compared with men. There was no trend in Hb concentration across age groups. Mean Hb was 4 g/dl lower (10·0 g/dl) among those with moderate/severe anaemia compared with those who were not anaemic (14·0 g/dl).

**Table 2:** Mean and 95 % CI for Hb measurement by age/sex category

	Age (years)	Mean (g/dl)	95 % CI
Study sample		13·2	13·1, 13·4
Males	15–19	13·5	13·1, 13·9
	20–29	13·4	13·1, 13·8
	30–39	14·1	13·7, 14·5
	40–49	13·7	13·4, 14·1
Females	15–19	12·8	12·2, 13·4
	20–29	12·7	12·4, 13·0
	30–39	12·8	12·5, 13·1
	40–49	12·8	12·4, 13·2
Anaemic (moderate/severe)		10·0	9·7, 10·2
Anaemic (mild)		11·8	11·7, 11·9
Not-anaemic		14·0	13·9, 14·2

Values expressed in g/dl.

**Fig. 1:**
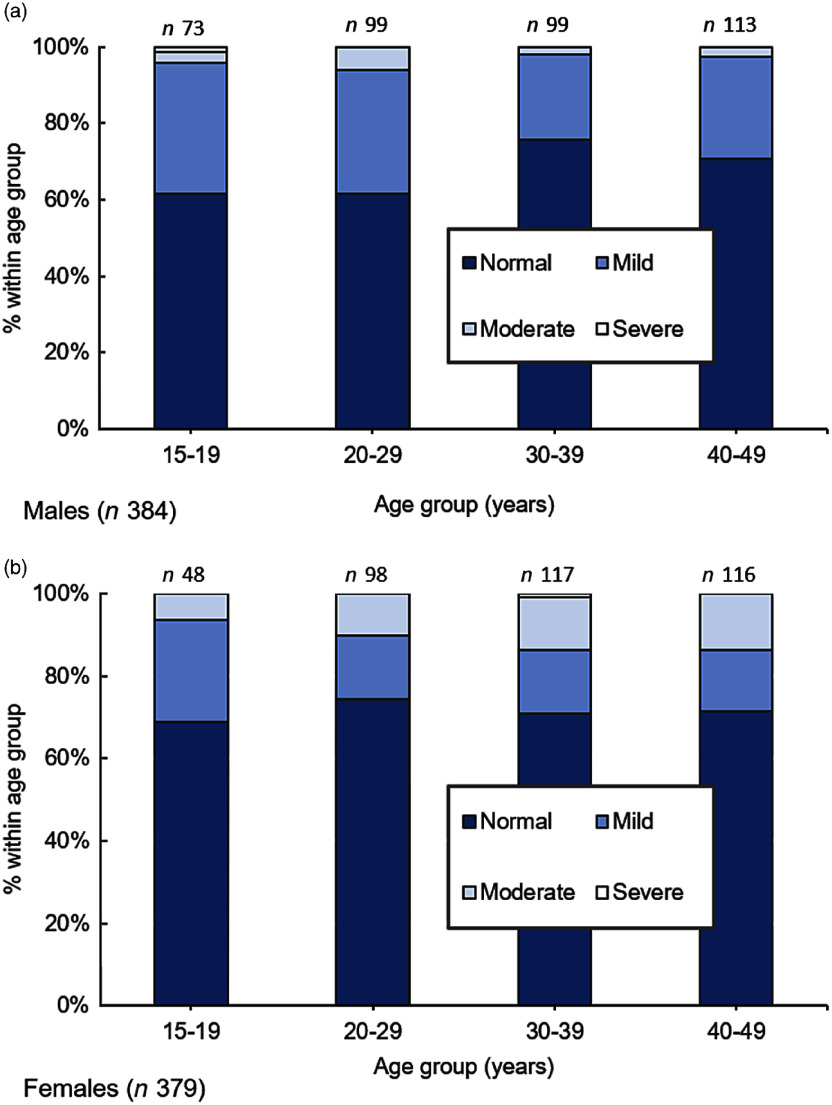
Prevalence (%) of anaemia by age and sex group

The prevalence of anaemia by age/sex and crude associations between demographic and nutritional factors with anaemic status are presented in Fig. 1 and Table 3. Almost one-third (30·2 %) of the study population was anaemic. The highest prevalence of anaemia was observed among 20–29-year-old males (38·4 %) and village Rajon (40·5 %). Moderate anaemia was more common among females in all age groups. The risk factor with strongest positive association with anaemia was underweight status (OR = 2·46, 95 % CI = 1·06, 5·72, *P* = 0·04). Prevalence of anaemia varied between 19·4 % and 40·5 % in villages, and there was a significant negative association between residing in Moyosari compared with Topengan with anaemic status (OR = 0·49, 95 % CI = 0·31, 0·78, *P* < 0·05). Consuming any category of soybean-based food had a negative association with anaemia, however only the tofu only category had a statistically significant association (OR = 0·54, 95 % CI = 0·31, 0·95, *P* < 0·05). Diet diversity had a positive association with anaemia (OR = 1·23, 95 % CI = 0·90, 1·69, *P* = 0·19), although no association between education levels or STH infection was found with anaemia.

**Table 3:** Bivariate analysis of the associations between socio-demographic and nutrition factors with anaemia

		Anaemic *n*	%	OR	95 % CI	*P*-value
Study sample		231	30·4	N/A		N/A
Village	Topengan	59	32·8	–		–
	Moyosari	42	19·4	**0·49**	**0·31, 0·78**	**0·002**
	Rajon	66	40·5	1·40	0·90, 2·17	0·14
	Losari	64	31·1	0·92	0·60, 1·42	0·72
Education	No school	16	27·1	–		–
	Elementary	97	29·8	1·14	0·61, 2·13	0·67
	Junior secondary	71	30·9	1·20	0·63, 2·27	0·58
	Senior secondary	39	33·6	1·36	0·68, 2·72	0·38
	College or higher	8	22·2	0·77	0·29, 2·03	0·60
Anthropometry	Normal	133	30·7	–		–
	Underweight	12	52·2	**2·46**	**1·06, 5·72**	**0·04**
	Overweight/obese	86	27·7	0·87	0·63, 1·20	0·38
Soil-transmitted helminth infection	Yes	22	29·3	0·97	0·57, 1·66	0·92
	No	129	29·9	–		–
Dietary diversity	Low	138	28·5	–		
	High	93	33·0	1·23	0·90, 1·69	0·19
High Fe food consumption	No soybean	56	36·8	–		–
	Tempeh only	92	29·5	0·72	0·48, 1·08	0·11
	Tofu only	24	24·0	**0·54**	**0·31, 0·95**	**0·03**
	Tempeh and Tofu	59	29·2	0·71	0·45, 1·11	0·13

Odds ratios in bold typeface are statistically significant at *P* < 0·05.

Three participants excluded due to missing data on at least one covariate.

Data for STH infection status were incomplete due to loss-to-follow-up in the overarching sanitation improvement intervention and as such was not included in the logistic regression model to retain statistical power.

Results from binary logistic regression analysis for anaemic status are presented in Table 4. Variables in model 1 were adjusted for age, sex and village. After adjusting for these variables, the adjusted OR for anaemia for soybean consumption categories did not change noticeably from crude OR. The significance of the association between high diet diversity and anaemia dropped from *P* = 0·19 to *P* = 0·08, and the OR for underweight rose to 2·75 (95 % CI = 1·13, 6·69, *P* < 0·05). After mutually adjusting for all variables in Model 2, the association of tofu only with anaemia only differed slightly (AOR = 0·53) and the association with underweight reduced slightly (AOR = 2·59). High diet diversity became statistically significant (AOR = 1·40, 95 % CI = 1·00, 1·97, *P* < 0·05). Whilst not statistically significant at *P* < 0·05, the associations between tempeh only and tempeh and tofu with anaemia were negative (AOR = 0·71, 95 % CI = 0·46, 1·09, *P* = 0·12, and AOR = 0·67, 95 % CI = 0·42, 1·06, *P* = 0·09 respectively).

**Table 4:** Binary logistic regression for anaemic status

		Model 1*			Model 2†		
		AOR	95 % CI	*P*	AOR	95 % CI	*P*
Soybean consumption	No soybean (*n* 150)						
	Tempeh only (*n* 310)	0·74	0·49, 1·12	0·16	0·71	0·46, 1·09	0·12
	Tofu only (*n* 99)	**0·55**	**0·31, 0·98**	**0·04**	**0·53**	**0·30, 0·95**	**0·03**
	Tempeh and Tofu (*n* 201)	0·68	0·43, 1·08	0·10	0·67	0·42, 1·06	0·09
Anthropometry	Normal (*n* 544)						
	Underweight (*n* 35)	**2·75**	**1·13, 6·69**	**0·03**	**2·59**	**1·05, 6·35**	**0·04**
	Overweight/obese (*n* 181)	1·07	0·75, 1·52	0·73	1·03	0·72, 1·48	0·87
Diet diversity	Low (*n* 477)						
	High (*n* 283)	1·34	0·96, 1·86	0·08	**1·40**	**1·00, 1·97**	**0·05**

Total *n* 760.

Odds ratios in bold typeface are statistically significant at *P* < 0·05.

* Adjusted for age, sex and village.

† Adjusted for age, sex, village, soybean consumption, anthropometry and diet diversity.

## Discussion

Results from our study demonstrated that anaemia is prevalent among 15–49-year-old villagers in rural Central Java, and soybean consumption is associated with a reduced risk of anaemia. Our results suggest that routine assessment of anaemia prevalence in Indonesia is critical given the moderate prevalence we observed, and soybean consumption, in particular tofu, could be considered in anaemia-prevention programmes in Indonesia.

The prevalence of anaemia in our study population was 30·2 % (Table 3), which is categorised as moderate public health significance according to the WHO^(35)^. Among young males and one particular village anaemia prevalence is almost or at 40 % (Fig. 1(a), Table 3), indicating severe public health significance. Whilst estimates of anaemia prevalence are influenced by the demographics surveyed, our results are similar to the 2008 estimate of 21·1 % among all individuals in the Indonesian Family Life Survey 4^(6)^. Among pregnant women in Indonesia, the prevalence of anaemia has actually steadily increased from 40·2 % in 2010 to 44·2 % in 2019^(5)^. Thus, strengthened interventions to target anaemia in Indonesia are needed. Updated nation-wide estimates of anaemia prevalence in Indonesia are required for greater understanding of the current situation. Anaemia prevalence was highest among young males whilst we did not quantify smoking status, may be explained by higher rates of smoking in this demographic^(41)^.

We found that soybean consumption is associated with reduced risk of anaemia after adjusting for BMI, diet diversity, STH infection, age, sex and village. Soybean consumption may reduce the risk of anaemia as it is naturally high in Fe^(34)^. The strongest association was in the ‘Tofu only’ category (AOR = 0·53, 95 % CI = 0·30, 0·95, *P* < 0·05, Table 4). These results are similar to a study among Chinese adults where among women, high consumption of tofu (derived from soybean) compared with low consumption was associated with reduced anaemia risk (AOR = 0·31, 95 % CI = 0·20, 0·47)^(32)^. Furthermore, authors of this study found that the odds of anaemia reduced with increasing tofu intake, suggesting a dose–response relationship. High Fe content of soybean^(29)^ is likely the determining factor in lowering the risk of IDA as it has been demonstrated among American women that Fe is absorbed from soybean in a clinical study^(34)^. An Indonesian study demonstrating that Fe-fortified tempeh consumption for 10 d was associated with a significant increase in serum Hb levels (increase = 0·52 g/dl, *P* < 0·05)^(42)^ supports our results and the idea that soybean consumption would be protective to IDA by causing an increase in Hb levels. Interestingly, despite consumption of tofu being greatly lower than tempeh (Table 1), the protective association was stronger for tofu (Table 4). This might be due to the higher Fe content in cooked tofu (4·87 mg/100 g) compared with tempeh (2·13 mg/100 g)^(43)^. Neither contain the Fe absorption enhancer vitamin C, and so consumption of foods high in vitamin C in tandem with tofu/tempeh would be expected to have the greatest impact on Fe and Hb levels. It might also be possible that by containing more fibre, tempeh (which is made from whole soybeans as opposed to tofu which is a soybean curd) leads to slower Fe absorption and therefore reduced impact on anaemia risk.

The association between underweight and anaemia (AOR = 2·59, 95 % CI = 1·05, 6·35, Table 4) is consistent with other studies in south-east Asia where it is thought that underweight women are malnourished and therefore more likely to suffer IDA^(19)^. We did not observe an association between STH infection and anaemia (AOR = 0·97, 95 % CI = 0·57, 1·66, Table 3) despite other studies finding a significant association^(24)^. It is possible that infection intensity and duration were not sufficient to cause anaemia among infected participants, but also due to insufficient statistical power. The notion that there was an association between high diet diversity and anaemia is counterintuitive. There may be other confounding variables not adjusted for in this study, and this relationship requires further investigation.

Encouraging soybean consumption as part of a staple diet could be effective in preventing anaemia when coupled with Fe supplementation. This is because whilst issues of non-compliance and inconsistent use of Fe supplementation in Indonesia have been reported^(11)^, the consumption of soybean in our study was almost 80 %, which was expected given it is a local staple (Table 1). Whilst Fe fortification of wheat flour has been mandatory in Indonesia since 2001^(44)^, consumption of wheat flour products among adults in our study was very low; the dominant carbohydrate source was white rice^(45)^. Only recently has Fe fortification of rice programmes began in Indonesia, but with limited success in increasing serum Hb and ferritin levels^(44)^. The added health benefits of soybean should also be mentioned, including a recent study that found that a drink made from soybean protein had anti-inflammatory properties among pregnant women in Malaysia^(46)^.

Our study has several limitations. We did not report the consumption of soybean as a continuous numeric variable due to uncertainty in estimating the amounts of soybean products present in composite dishes. This could cause our association between soybean and anaemia to be underestimated as greater consumption of soybean which would provide greater Fe intake would be assumed to provide an even greater protective association with anaemia. Because the food recall was only relevant to a single day, due to chance there may be individuals who regularly consume soybean but not in the 24 h prior to the data collection. This would bias the association towards the null. However, *post hoc* analysis of a FFQ that reports historical consumption among the same study population as part of the overarching study revealed a negative association between soybean and anaemia. This indicated that the association reported from the 24-h food recall is reliable. Furthermore, some socio-economic and other health conditions that may associate with anaemia (e.g. HIV, inflammation) were not included in the model and we did not measure smoking behaviour which could affect the associations observed. The high prevalence of anaemia among young males may be driven by smoking. However, whilst these variables may correlate with anaemia, they most likely do not correlate with soybean consumption in this study context, therefore we do not expect that adjusting for these variables would have great effect on the association between soybean consumption and anaemia. We did not record pregnancy status among women and as such we cannot report our association directly among pregnant women. Measuring blood Hb concentration is not as accurate as recording blood ferritin levels from a serum blood sample to assess for Fe-deficient anaemia, nor does it permit the possibility to assess non-Fe deficient anaemia.

## Conclusion

Results from our study indicate that anaemia prevalence is relatively consistent with reported nation-wide data. Soybean consumption, in particular tofu, was associated with a lower risk of anaemia. Soybean, unlike other meat or animal products that might be encouraged to prevent anaemia, is considerably affordable in Indonesia. Furthermore, it is a culturally acceptable food with other added health and dietary benefits. Consumption of soybean was high, and future research should evaluate the quantity of soybean consumed and its association with anaemia. Encouraging the consumption of soybean products among demographics at high risk of anaemia may work in tandem with existing interventions to prevent anaemia in Indonesia.
